# Runx1 protects against the pathological progression of osteoarthritis

**DOI:** 10.1038/s41413-021-00173-x

**Published:** 2021-12-07

**Authors:** Chenchen Zhou, Yujia Cui, Yueyi Yang, Daimo Guo, Demao Zhang, Yi Fan, Xiaobing Li, Jing Zou, Jing Xie

**Affiliations:** 1 State Key Laboratory of Oral Diseases, West China Hospital of Stomatology, Sichuan University, Chengdu, China; 2 National Clinical Research Center for Oral Diseases, Sichuan University, Chengdu, China; 3 Department of Pediatric Dentistry, West China Hospital of Stomatology, Sichuan University, Chengdu, China

**Keywords:** Bone, Metabolic syndrome

## Abstract

Runt-related transcription factor-1 (Runx1) is required for chondrocyte-to-osteoblast lineage commitment by enhancing both chondrogenesis and osteogenesis during vertebrate development. However, the potential role of Runx1 in joint diseases is not well known. In the current study, we aimed to explore the role of Runx1 in osteoarthritis induced by anterior cruciate ligament transaction (ACLT) surgery. We showed that chondrocyte-specific Runx1 knockout (*Runx1*^*f/f*^*Col2a1-Cre*) aggravated cartilage destruction by accelerating the loss of proteoglycan and collagen II in early osteoarthritis. Moreover, we observed thinning and ossification of the growth plate, a decrease in chondrocyte proliferative capacity and the loss of bone matrix around the growth plate in late osteoarthritis. We overexpressed Runx1 by adeno-associated virus (AAV) in articular cartilage and identified its protective effect by slowing the destruction of osteoarthritis in cartilage in early osteoarthritis and alleviating the pathological progression of growth plate cartilage in late osteoarthritis. ChIP-seq analysis identified new targets that interacted with Runx1 in cartilage pathology, and we confirmed the direct interactions of these factors with Runx1 by ChIP-qPCR. This study helps us to understand the function of Runx1 in osteoarthritis and provides new clues for targeted osteoarthritis therapy.

## Introduction

Runt-related transcription factor-1 (Runx1), which is also known as core-binding factor α 2 or acute myeloid leukemia 1 (AML1), is known for its vital role in hematopoiesis and blood malignancies.^1^ In humans, the RUNX1 gene is one of the most common targets of genetic alterations in acute leukemia.^2^ In newborn mice, conventional knockout of Runx1 leads to the loss of hematopoietic capacity, and individuals cannot survive the early embryonic stage.^3^ In adult mice, conditional knockout (CKO) of Runx1 in the hematopoietic lineage directly causes myeloid malignancies, such as myelodysplastic syndrome, myeloproliferative neoplasm-like disease, and acute myeloid leukemia (AML).^4,5^ As a master regulatory transcription factor, Runx1 has been implicated in a wide range of organ development processes and disease occurrence beyond the hematopoietic system.^6^ In skeletal development, Runx1 plays a fundamental role in the lineage determination of progenitor cells in the periosteum, calvarial sutures and perichondrium,^7^ and thus contributes to skeletogenesis in the early stage.^8^ In vitro, Luo et al. showed that Runx1 regulated the osteogenic differentiation of bone marrow stem cells (BMSCs) by inhibiting adipogenesis through the Wnt/β-catenin pathway.^9^ Wang et al. found that overexpression of Runx1 induced BMSCs to undergo chondrogenic differentiation.^10^ In vivo, Kimura et al. revealed that Runx1 was an important regulatory factor in sternal morphogenesis due to mineralization impairment induced by CKO of Runx1 in paired-related homeobox transcription factor-1 (Prrx1)- and *Col2a1-Cre* mice.^11^ We previously reported that Runx1 promoted chondrocyte-to-osteoblast lineage commitment^12^ and showed that Runx1 was a prerequisite for murine osteoblast differentiation and final bone formation.^13^ Although the importance of Runx1 in skeletal development has been gradually elucidated, its role in cartilage diseases such as osteoarthritis (OA) remains unclear.

Runx1 is a core-binding factor that consists of Cbf-alpha (Cbfα) and Cbf-beta (Cbfβ). Cbfβ is encoded by a single gene, but Cbfα is encoded by the runt-related transcription factors (Runxs) Runx1, 2 and 3.^14^ Runx family members have distinct spatial-temporal and tissue-specific expression patterns and show distinct and nonredundant biological functions,^15^ although they are highly conserved in the runt domain and bind to the consensus DNA sequence through the dimerization of Cbfβ.^16^ Runx1 is well characterized and is definitive in hematopoiesis and hematological malignancies, such as AML (thus, Runx1 is also called AML1), but its complicated implications in diverse signaling pathways and cellular mechanisms determines its potent role as a master-regulator transcription factor.^17^ Runx2 is best known for its requisite role in osteoblast differentiation and skeletal development and is an osteogenic marker.^18^ Runx3 has been shown to play a role in dorsal root ganglia proprioceptive neuron development,^19^ and it may also interact with Runx2 to regulate chondrocyte development.^20^ In skeletal development, we previously demonstrated the role of Runx1 in the osteoblast lineage,^13^ chondrocyte-to-osteoblast commitment,^12^ and the activation of multiple signaling pathways.^21^ However, the importance of Runx1 in disease occurrence and progression needs to be further explored.

OA, which is one of the most common joint diseases, is a leading cause of disability and is becoming a worldwide burden with economic costs.^22^ Despite its increasing significance, no approved therapies can prevent the progression of OA, and no consensus has been reached on the pathological mechanism of OA due to its multiple etiologies.^23^ In the current study, we examined the important role of Runx1 in OA and its potential therapeutic effect in a mouse model from a genetics perspective.

## Results

### Runx1 knockout aggravates cartilage destruction in osteoarthritis

**Fig. 1:**
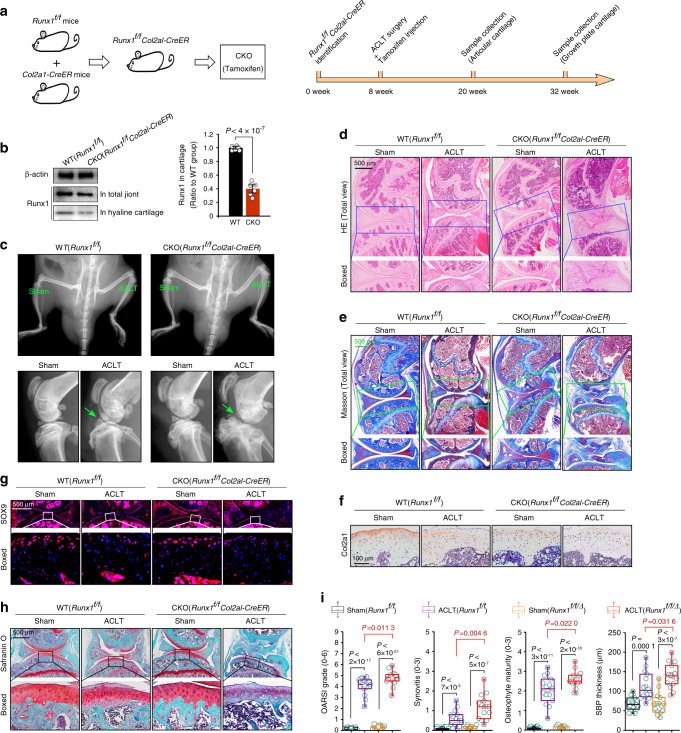
Runx1 knockout aggravates articular cartilage destruction in an ACLT-induced mouse OA model. **a** Schematic diagram showing the generation of Runx1-knockout mice and the detailed sample collection procedure. We achieved chondrocyte-specific Runx1 knockout by intraperitoneal tamoxifen injection at 3–5 days after recovery from ACLT surgery. We performed intraperitoneal tamoxifen injection twice per week. After 2 weeks of injections, cartilage genotyping was performed to determined whether Runx1 knockout was achieved. **b** Western blotting and quantification showing the expression of Runx1 in the total joint and articular cartilage of Runx1-knockout mice. **c** In vivo imaging showing the joint destruction in Runx1-knockout mice at 12 weeks after ACLT surgery. **d** H&E staining showing pathological changes in articular cartilage in Runx1-knockout mice at 12 weeks after ACLT surgery. **e** Masson staining showing pathological changes in articular cartilage in Runx1-knockout mice at 12 weeks after ACLT surgery. **f** IHC staining showing the expression of Col2a1 in articular cartilage in Runx1-knockout mice at 12 weeks after ACLT surgery. **g** Immunofluorescence showing the expression of SOX9 in articular cartilage in Runx1-knockout mice at 12 weeks after ACLT surgery. **h** Safranin O staining showing the loss of proteoglycan in articular cartilage in Runx1-knockout mice at 12 weeks after ACLT surgery. **i** The degree of experimental mouse OA was evaluated by scoring cartilage destruction (Osteoarthritis Research Society International (OARSI) grade), synovitis, osteophyte maturity, and subchondral bone plate (SBP) thickness (suggestive of sclerosis). *Runx1*^*f/f/Δ*^ Runx1 knockout. These results are based on at least three independent experiments (*n* = 3). The standard Mann–Whitney *U* test was used for the OARSI grade, synovitis, osteophyte maturity and SBP thickness, and the data in **i** (right) are shown as box (from 25%, 50% to 75%) and whisker (minimum to maximum values) plots. All significance data presented in **b** and **i** are based on two-tailed Student’s *t*-tests

We crossed *Runx1*^*f/f*^ mice with *Col2a1-CreER*^*T*^ mice and obtained *Runx1*^*f/+*^*Col2a1-CreER*^*T*^ mice. We then inbred *Runx1*^*f/+*^*Col2a1-CreER*^*T*^ mice (heterozygotes) to generate *Runx1*^*f/f*^*Col2a1-CreER*^*T*^ mice (homozygotes). As we aimed to explore the role of Runx1 in the pathological process of OA in cartilage, after establishing an OA model by anterior cruciate ligament transaction (ACLT) surgery,^24^ we achieved CKO of Runx1 in cartilage by intraperitoneal injection of tamoxifen (injection was performed ~3–5 days after recovery from ACLT surgery). We collected knee joint samples at 12 weeks post surgery to examine changes in articular cartilage (when OA phenotype of articular cartilage is obvious during early OA) and 24 weeks post surgery to examine changes in growth plate cartilage (when the OA phenotype of subchondral bone is comparatively strong during late OA) (Fig. 1a). We first examined the CKO efficiency at 5 days after tamoxifen injection (Figs. 1b and S1). The results showed that Runx1 expression was significantly reduced in both articular cartilage and growth plate cartilage (Fig. S1). At 12 weeks after ACLT surgery, we examined the typical OA phenotype of the knee joint by in vivo imaging due to the indistinct shape of joint tissue (green arrow). In the Runx1-CKO group, joint deformation and tissue fragments were easily observed (Fig. 1c). We used histology to confirm joint destruction. H&E staining (Fig. 1d) and Masson staining (Fig. 1e) showed that joint destruction was more serious in the Runx1-CKO OA group than in the WT OA group. We then examined the expression of the cartilage markers collagen II (Col2a1) and SOX9. The results showed that the expression levels of Col2a1 and SOX9 were much lower in the Runx1-CKO OA group than in the WT OA group (Fig. 1f, g). We next used safranin O staining to examine changes in proteoglycans in articular cartilage (Fig. 1h). The results indicated more proteoglycan loss in the Runx1-CKO OA group than in the WT OA group, even if there was little change in cartilage morphology. Finally, we evaluated the OA severity rating in the mouse ACLT-OA model by scoring cartilage destruction (OARSI grade, 0–6), synovitis (0–3), osteophyte maturity (0–3), and subchondral bone plate (SBP) thickness (Fig. 1i) and found more serious knee joint destruction in the Runx1-CKO OA groups than in the WT OA groups. Taken together, these results suggested that Runx1 knockout accelerated the pathological changes in the entire knee joint and the destruction of articular cartilage.

### Runx1 knockout affects the pathological changes in growth plate cartilage in the late stage of osteoarthritis

**Fig. 2:**
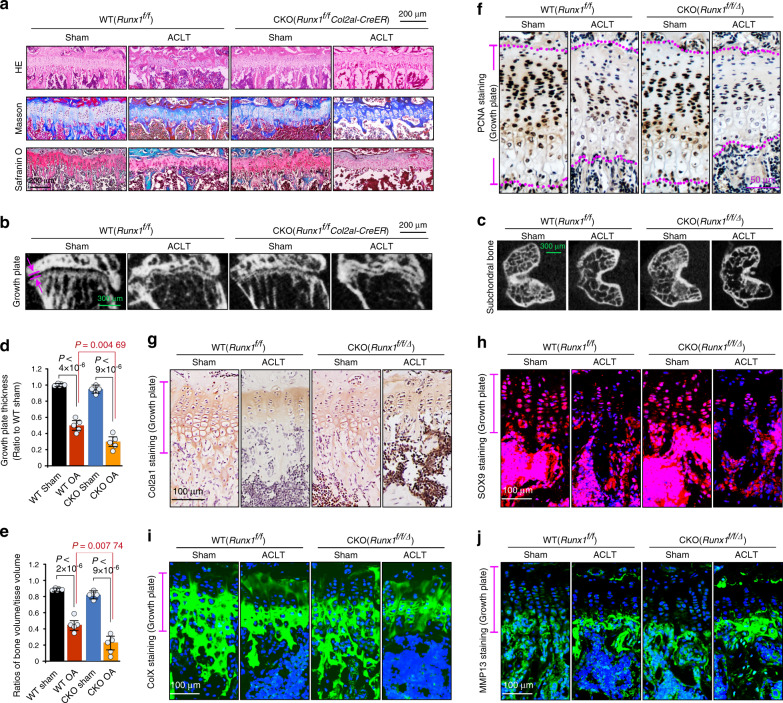
Runx1 knockout affects the pathological changes in growth plate cartilage. **a** Histological staining, including H&E, Masson, and safranin O, showing the changes in growth plate cartilage in Runx1-knockout mice at 24 weeks after ACLT surgery. **b** μ-CT showing the thickness change in growth plate cartilage in Runx1-knockout mice at 24 weeks after ACLT surgery. **c** μ-CT showing the changes in subchondral cancellous bone in Runx1-knockout mice at 24 weeks after ACLT surgery. **d** Quantitative analysis of growth plate width (thickness) in **b** Runx1 knockout mice at 24 weeks post ACLT surgery. **e** Quantitative analysis of BV/TV in **c** in Runx1 knockout mice at 24 weeks post ACLT surgery. **f** IHC staining showing the proliferative capacity of chondrocytes by characterizing PCNA expression in the growth plate in Runx1-knockout mice at 24 weeks after ACLT surgery. **g** IHC staining showing the changes in Col2a1 in the growth plate in Runx1-knockout mice at 24 weeks after ACLT surgery. **h** Immunofluorescence staining showing the changes in SOX9 in the growth plate in Runx1-knockout mice at 24 weeks after ACLT surgery. **i** Immunofluorescence staining showing the changes in ColX in the growth plate in Runx1-knockout mice at 24 weeks after ACLT surgery. **j** Immunofluorescence staining showing the changes in MMP13 in the growth plate in Runx1-knockout mice at 24 weeks after ACLT surgery. All these results above are based on at least three independent experiments (*n* = 3). All significance data presented in **d** and **e** are based on two-tailed Student’s *t*-tests. *Runx1*^*f/f/∆*^ Runx1 knockout

To further examine the effect of Runx1 knockout at the late stage of OA, we analyzed pathological changes in growth plate cartilage in the femur at 24 weeks after ACLT surgery. The histology results (Fig. 2a) showed that increased ossifying matrix was lost around growth plate cartilage (above and below), and chondrocyte clusters tended to be directly exposed to the medullary cavity. μ-CT analysis showed that the thickness of growth plate cartilage was significantly reduced (Fig. 2b), and trabecular bone in the second ossification zone was notably lost (Fig. 2c) in the Runx1-knockout OA group relative to the WT OA group. Quantitative analysis of growth plate thickness (Fig. 2d) and the BV/TV ratio (Fig. 2e) confirmed these results. IHC staining was used to examine the chondrocyte proliferative capacity with PCNA (Fig. 2f) and showed that increased numbers of chondrocytes in the Runx1-knockout OA group lost their proliferative capacity. We then measured the expression of Col2a1 by IHC (Fig. 2g) and SOX9 by IF (Fig. 2h). The results showed that the expression of Col2a1 and SOX9 was reduced in the Runx1-knockout OA groups relative to the WT OA groups. We next examined the expression of collagen X (ColX, Fig. 2i), which is a marker of hypertrophic cartilage, and found that the distribution of ColX was limited to chondrocyte clusters, which might be due to the serious loss of the surrounding ossifying matrix. We finally measured the expression of MMP13 in hypertrophic cartilage growth plates (Fig. 2j), and the results showed more accumulation of MMP13 in the Runx1-knockout OA group than in the WT OA group. These results indicated that Runx1 knockout impacted the pathological progression of growth plate cartilage in the late stage of OA.

### Runx1 overexpression slows cartilage destruction in osteoarthritis

**Fig. 3:**
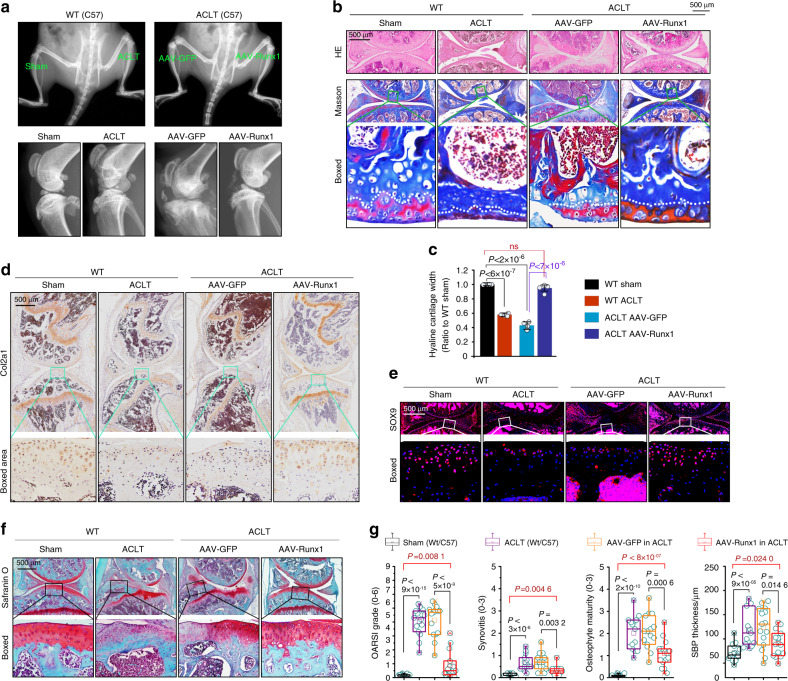
AAV-Runx1 overexpression protects against cartilage destruction in an ACLT-induced mouse OA model. **a** In vivo imaging showing that overexpression of Runx1 in chondrocytes protects against cartilage destruction in an ACLT-induced mouse OA model. **b** Histological staining including HE, Masson, and safranin O showing the effects of AAV-Runx1 overexpression in articular cartilage at 12 weeks post ACLT surgery. **c** Quantitative analysis indicating the changes in mature articular cartilage thickness in **b**. **d** IHC staining showing the expression of Col2a1 in AAV-Runx1-overexpressing articular cartilage at 12 weeks post ACLT surgery. **e** Immunofluorescence staining showing the changes in SOX9 in AAV-Runx1-overexpressing articular cartilage at 12 weeks post ACLT surgery. **f** Safranin O staining showing the changes in proteoglycans in AAV-Runx1-overexpressing articular cartilage at 12 weeks post ACLT surgery. **g** Cartilage protection effects in AAV-Runx1-overexpressing articular cartilage were evaluated by OARSI grade, synovitis, osteophyte maturity, and SBP thickness. These results are based on at least three independent experiments (*n* = 3). The standard Mann–Whitney *U* test was used for the OARSI grade, synovitis, osteophyte maturity and SBP thickness, and the data in **g** are shown as box (from 25%, 50% to 75%) and whisker (minimum to maximum values) plots. All significance data presented in **c** and **g** are based on two-tailed Student’s *t*-tests

We established a Runx1 overexpression model in vivo by using adeno-associated virus (AAV). After 3–5 days of recovery from ACLT surgery, AAV-Runx1 was injected into the articular cavity (AAV-GFP was used as the sham control). At 12 weeks after ACLT surgery, we first examined AAV-Runx1 overexpression in articular cartilage (frozen section, GFP, Fig. S2). In vivo imaging showed that AAV-Runx1 overexpression protected against the destruction of articular cartilage (Fig. 3a), although partial tissue fragments were present. H&E and Masson staining (Fig. 3b) showed that the AAV-Runx1 group retained the general integrity of articular cartilage relative to that in the WT OA group and AAV-GFP OA group. The thickness of the mature chondrocyte region was close to that of the WT sham group. Quantitative analysis of the thickness confirmed these results (Fig. 3c). We then examined the effect of AAV-Runx1 on Col2a1 and SOX9 expression. IHC staining showed that the expression of Col2a1 was significantly retained relative to that in the WT OA group and AAV-GFP OA group, although Col2a1 expression was relatively lower than that in the WT sham group (Fig. 3d). The analysis of SOX9 showed the same trend as that of Col2a1 (Fig. 3e). We next performed safranin O staining to examine changes in cartilage proteoglycan (Fig. 3f), and the results showed that the AAV-Runx1 group had preserved integrity of articular cartilage and that more proteoglycan was retained than the WT OA group and AAV-GFP OA group. We finally evaluated the OA severity rating to show the effect of AAV-Runx1 (Fig. 3g). These results showed that AAV-Runx1 overexpression alleviated the progression of OA and preserved the integrity of the knee joint.

### Runx1 overexpression alleviates the pathological progression of growth plate cartilage in the late stage of osteoarthritis

**Fig. 4:**
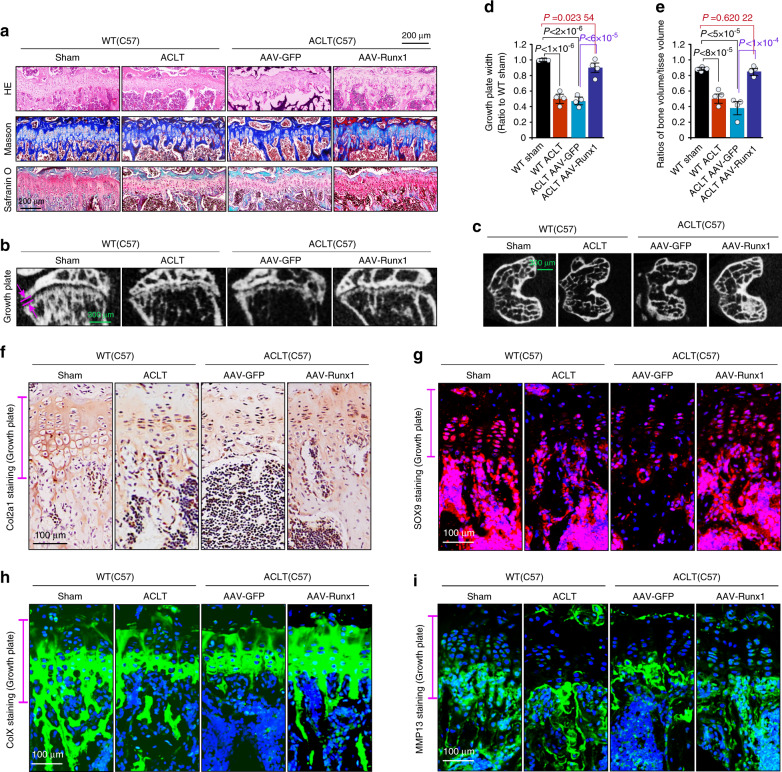
AAV-Runx1 overexpression alleviates the pathological changes in growth plate cartilage. **a** Histological staining, including H&E, Masson, and safranin O, showing the effects of AAV-Runx1 overexpression on growth plate cartilage at 24 weeks after ACLT surgery. **b** μ-CT results showing changes in the thickness of growth plate cartilage in AAV-Runx1-overexpressing articular cartilage at 24 weeks after ACLT surgery. **c** μ-CT results showing the changes in subchondral cancellous bone in AAV-Runx1-overexpressing articular cartilage at 24 weeks after ACLT surgery. **d** Quantitative analysis of the thickness (width) in **b** in growth plate cartilage in AAV-Runx1-overexpressing mice. **e** Quantitative analysis of BV/TV in **c** in AAV-Runx1-overexpressing mice at 24 weeks after ACLT surgery. **f** IHC staining showing the changes in Col2a1 in the growth plate in AAV-Runx1-overexpressing articular cartilage at 24 weeks after ACLT surgery. **g** Immunofluorescence staining showing the changes in SOX9 in the growth plate in AAV-Runx1-overexpressing articular cartilage at 24 weeks after ACLT surgery. **h** Immunofluorescence staining showing the changes in ColX in the growth plate in AAV-Runx1-overexpressing articular cartilage at 24 weeks after ACLT surgery. **i** Immunofluorescence staining showing the changes in MMP13 in the growth plate in AAV-Runx1-overexpressing articular cartilage at 24 weeks after ACLT surgery. These results are based on at least three independent experiments (*n* = 3). All significance data presented in **d** and **e** are based on two-tailed Student’s *t*-tests

We also examined the effect of AAV-Runx1 overexpression on the pathological progression of growth plate cartilage during the late stage of OA. At 24 weeks after ACLT surgery, we examined the changes in growth plate cartilage by histology. The H&E, Masson, and safranin O staining results (Fig. 4a) showed that AAV-Runx1 overexpression preserved the thickness of growth plate cartilage relative to that in the WT OA group and the AAV-GFP OA group. μ-CT analysis further showed the preserved width (thickness) of growth plate cartilage in the AAV-Runx1 overexpression group relative to that in the WT OA group and the AAV-GFP OA group (Fig. 4b), while trabecular bone in the subchondral bone region was also retained (Fig. 4c). Although the growth plate width in the AAV-Runx1 overexpression group did not reach that in the WT sham group (Fig. 4d), the trabecular bone number in the subchondral bone region was the same as that in the WT sham group (*P* > 0.05, Fig. 4e). We then measured the expression of Col2a1 by IHC (Fig. 4f) and SOX9 by IF (Fig. 4g) and found that expression was higher than that in the WT OA group and AAV-GFP OA group. IF staining for ColX showed that ColX was expressed throughout the entire hypertrophic cartilage area in the AAV-Runx1 overexpression group (Fig. 4h), indicating that hypertrophic cartilage was effectively preserved. Moreover, IF staining of MMP13 further confirmed AAV-Runx1-mediated preservation of hypertrophic cartilage in the pathological progression of OA (Fig. 4i).

### ChIP sequencing identified new gene targets in cartilage pathology

**Fig. 5:**
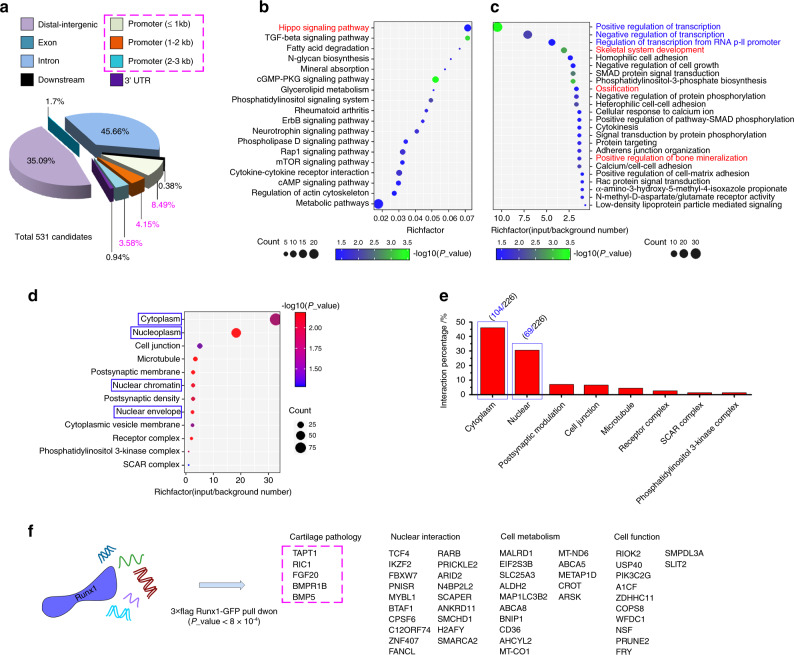
ChIP sequencing identified the key target genes in cartilage pathology. **a** Pie chart showing the total classification of target DNA fragments pulled down by the 3× Flag-Runx1-GFP tag. **b** KEGG analysis showing that the DNA fragments in the promoter region (≤3 kb) were enriched in 18 pathways. **c** Biological process (BP) GO analysis showing that the targets that interact with Runx1 had high proportions in the cytoplasm and nucleus. Red processes show targets related to cartilage pathology. Blue processes show targets in the nucleus. **d** Cellular component (CC) GO analysis showing the distributions of targets among chondrocyte components. **e** The specific proportions of targets that interact with Runx1 in chondrocytes. **f** Gene functional analysis indicating new targets that interact with Runx1 in chondrocytes in addition to the reported conventional binding genes

To confirm the mechanism of Runx1 in cartilage pathology, we aimed to identify potential gene targets that directly interact with the transcription factor Runx1. Thus, we first overexpressed Runx1 in chondrocytes with the lentivirus and performed ChIP to pull down all related DNA fragments. ChIP sequencing was used to examine all DNA fragments and showed that 16.22% appeared in the promoter region (Fig. 5a). We then performed KEGG pathway analysis of these DNA fragments in the promoter (Fig. 5b) and found that the results in chondrocytes were basically the same as those that had been previously reported in stem cells.^21^ However, importantly, we found that the Hippo signaling pathway was enriched in chondrocytes (red in Fig. 5b). We then performed functional GO analysis and identified the biological process (BP), and the results are shown in a bubble chart (Fig. 5c). The BPs in blue show the enrichment of nucleoproteins that interact with the transcription factor Runx1; moreover, the BPs in red further indicate the enrichment of targets in the skeletal system that interact with Runx1. We then showed the distribution of cellular components (CC) of these targets in a bubble chart (Fig. 5d) and found the specific distribution ratios of these targets (Fig. 5e). In addition to the conventional interacting factors (Table S1), we listed the specifically expressed targets in chondrocytes (Fig. 5f). Among them, most targets were involved in nuclear interactions, cell metabolism, and cell function, but five new targets (boxed in pink) were identified that were directly associated with cartilage pathology. These targets were transmembrane anterior posterior transformation 1 (TAPT1), protein RIC1 homolog (RIC1), fibroblast growth factor 20 (FGF20), bone morphogenetic protein receptor type 1B (BMPR1B), and bone morphogenetic protein 5 (BMP5).

### New verified pathogenic factors that interact with Runx1 in cartilage pathology

**Fig. 6:**
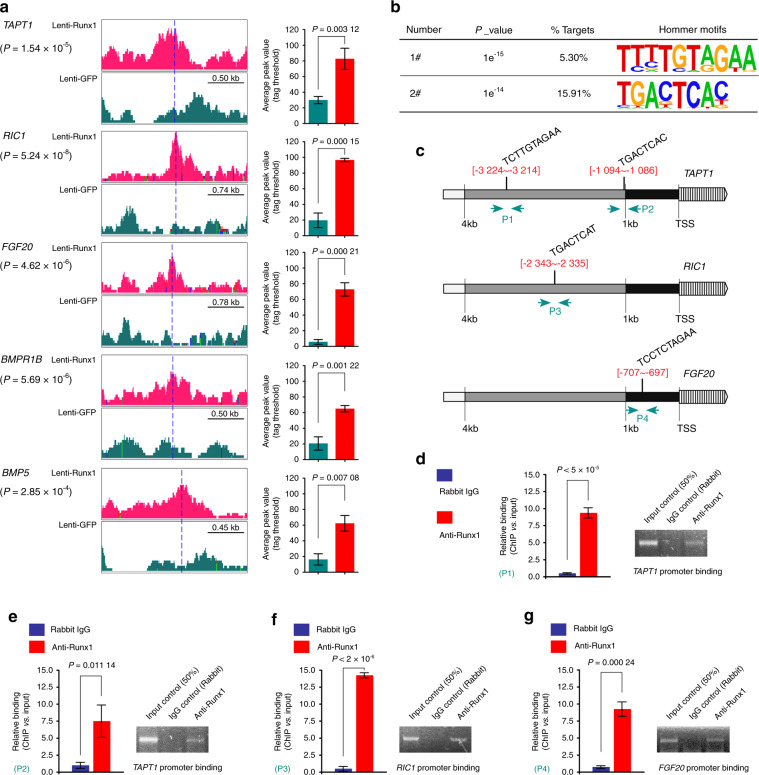
Detailed binding of Runx1 to the promoters of target genes in the context of chondrocyte pathology. **a** Peak values of target genes in chondrocyte pathology were identified by using IGV analysis. The specific average peak values are shown in the right histograms. **b** Motif analysis based on Runx1-ChIP-seq (hommer) identified two sequences at the promoter regions of these target genes in the context of chondrocyte pathology. **c** Bioinformatics analysis indicated the specific locations of these two sequences in the promoters of the *TAPT1*, *RIC1*, and *FGF20* genes. **d** ChIP-qPCR confirmed one binding site (−3 224 to −3 214) of Runx1 in the promoter of the *TAPT1* gene. Strip diagrams from electrophoretic gels based on ChIP-qPCR products confirmed this result. **e** ChIP-qPCR confirmed another binding site (−1 094 to −1 086) of Runx1 in the promoter of the TAPT1 gene. Strip diagrams from electrophoretic gels based on ChIP-qPCR products confirmed this result. **f** ChIP-qPCR confirmed the binding site (−2 343 to −2 335) of Runx1 in the promoter of the *RIC1* gene. Strip diagrams from electrophoretic gels based on ChIP-qPCR products confirmed this result. **g** ChIP-qPCR confirmed the binding site (−707 to −697) of Runx1 in the promoter of the *FGF20* gene. Strip diagrams from electrophoretic gels based on ChIP-qPCR products confirmed this result. These results are based on at least three independent experiments (*n* = 3). All significance data presented in **a**, **d**, **e**, **f**, and **g** are based on two-tailed Student’s *t*-tests

We performed further experiments on these five new targets. We found the peak values of these targets by ChIP-seq using IGV software and showed the average peak values in the Lenti-Runx1 group relative to the Lenti-GFP group (Fig. 6a). ChIP-seq was used to obtain 19 motifs based on all targets (Fig. S3) and identified two motifs (sequences: TCTTGTAGAA and TGACTCAC, Fig. 6b) in the promoters of *TAPT1*, *RIC1*, and *FGF20* after performing sequence alignment (no motifs were found in the promoters of *BMPR1B* and *BMP5*). We next identified the specific potential binding sites of these motifs in the promoters of *TAPT1*, *RIC1*, and *FGF20* (Fig. 6c). Finally, we designed specific primers (Supplementary Information File-II) and performed ChIP-qPCR to confirm these binding sites (Fig. 6d–g). Based on these results, we verified the four binding sites in the promoters of *TAPT1*, *RIC1*, and *FGF20*.

**Fig. 7:**
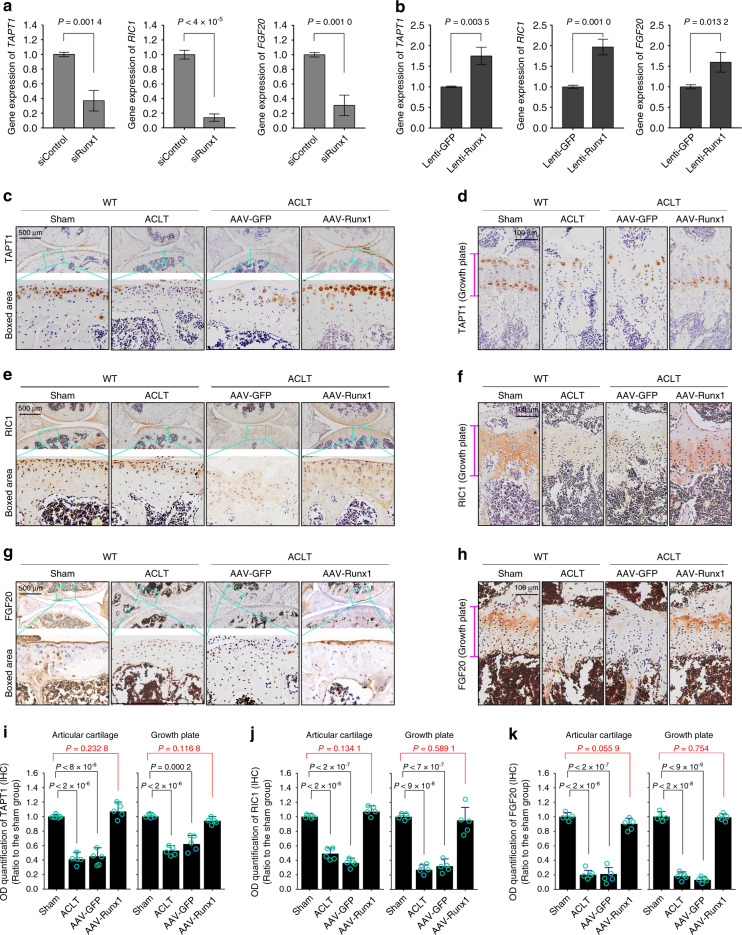
The expression of target candidates in cartilage. **a** qPCR results showing changes in *TAPT1*, *RIC1*, and *FGF20* in chondrocytes induced by siRunx1. **b** qPCR results showing changes in *TAPT1*, *RIC1*, and *FGF20* in chondrocytes induced by Runx1 overexpression. **c**, **d** IHC staining showing AAV-Runx1 overexpression-induced changes in TAPT1 expression in articular cartilage at 12 weeks after ACLT surgery (**a**) and the growth plate at 24 weeks after ACLT surgery (**b**). **e**–**f** IHC staining showing AAV-Runx1 overexpression-induced changes in RIC1 expression in articular cartilage at 12 weeks after ACLT surgery (**a**) and the growth plate at 24 weeks after ACLT surgery (**b**). **g**–**h** IHC staining showing AAV-Runx1 overexpression-induced changes in FGF20 expression in articular cartilage at 12 weeks after ACLT surgery (**a**) and the growth plate at 24 weeks after ACLT surgery (**b**). **i** Optical density quantitative analysis showing changes in TAPT1 expression in articular cartilage and the growth plate. **j** Optical density quantitative analysis showing changes in RIC1 expression in articular cartilage and the growth plate. **k** Optical density quantitative analysis showing changes in FGF20 expression in articular cartilage and the growth plate. These results are based on at least three independent experiments (*n* = 3). All significance data presented in **a**, **b**, **i**, **j**, and **k** are based on two-tailed Student’s *t*-tests

To confirm the gene expression of these three targets, we first used siRunx1 to examine gene changes (Fig. 7a). The results showed that the gene expression levels of *TAPT1*, *RIC1*, and *FGF20* were decreased at 12 h after siRNA interference. We then used lentivirus transfection of Runx1 to examine changes in these targets, which were all increased (Fig. 7b). To further confirm their expression at the protein level, we performed IHC staining (Fig. 7c–h). At 12 weeks after ACLT surgery, we found changes in the expression of TAPT1 (Fig. 7c), RIC1 (Fig. 7e) and FGF20 (Fig. 7g) in articular cartilage. AAV-Runx1 overexpression enhanced the expression of these three proteins to levels close to those of the sham groups. At 24 weeks after ACLT surgery, we measured the expression of TAPT1, RIC1, and FGF20 in the growth plate. The results indicated that in the late stage of OA, AAV-Runx1 overexpression preserved joint integrity and increased the levels of the target proteins TAPT1 (Fig. 7b), RIC1 (Fig. 7d), and FGF20 (Fig. 7f) to levels close to those of the sham groups. Further quantitative analysis also confirmed changes in the expression of TAPT1 (Fig. 7g), RIC1 (Fig. 7h), and FGF20 (Fig. 7i) in articular cartilage and the growth plate. Taken together, these results indicated that potential new pathogenic factors that interact with Runx1 were identified.

## Discussion

In the current study, we used *Col2a1-Cre* mice instead of *SOX9-Cre* mice to delete Runx1 in chondrocytes to verify the specific role of Runx1 in OA in adult mice. SOX9 plays a vital role in mesenchymal cell condensation and the initiation of progenitor cell differentiation into chondrocytes.^25^ The application of *SOX9-Cre* is more likely to be used in the context of cartilage development. *Col2a1-Cre* mice were used to track the effect of Runx1 mutations by characterizing changes in articular cartilage, growth palate cartilage and adjacent subchondral bone during the progression of OA. In the ACLT-OA mouse model, we found that the destruction of the whole joint was more serious in Runx1-knockout mice than in WT mice. The phenotypic changes in articular cartilage were aggravated due to the increased loss of collagen II and proteoglycan. The degree of ossification in the growth plate area increased due to the shortened growth plate spaces. In the AAV-Runx1 overexpression model, we observed the alleviation of joint destruction, articular cartilage damage, and growth plate deterioration. ChIP sequencing was used to identify new target genes that interact with Runx1 during the process of OA.

The direct role of Runx1 in skeletal development has recently been reported.^12,13,21^ In contrast, two other members of the Runx family (Runx2 and Runx3) were previously shown to be indispensable for chondrocyte hypertrophy.^18–20^ In Runx2-knockout mice, chondrocyte hypertrophy is largely decreased^20^ due to the critical role of Runx2 in the regulation of Indian hedgehog (Ihh) signaling, collagen X and vascular endothelial growth factors.^26^ Runx3, a potential mediator of neural cell differentiation, binds with Runx2 to regulate chondrocyte maturation and hypertrophy.^27^ Moreover, Runx2 and Runx3 double-knockout resulted in the complete loss of hypertrophic chondrocytes.^20^ Although Runx1 is a master regulatory transcription factor, the phenotypic difference caused by Runx1 deletion (using *Col2a1-Cre*) during postnatal development is much weaker than that of the other two members. Moreover, the phenotype of Runx1 deletion induced by *Col2a1-Cre* was not as strong as that in *Cre* mice, such as *Prrx1-Cre*,^11^ *Osx-Cre*,^13^ *Twist2-* and *Col1a1-Cre*.^21^ Recent reports have indicated that chondrocyte-specific Runx1 knockout by *Col2a1-Cre* results in impaired cartilage formation, decreased bone density, and an osteoporotic phenotype. Importantly, the vital role of Runx1 is to regulate chondrocyte-to-osteoblast lineage commitment and promote endochondral bone formation by enhancing both chondrogenesis and osteogenesis.^13,21^ In this study, we elucidated the role of Runx1 in the progression of OA from the aspect of cartilage pathology.

As a multifunctional transcription factor, Runx1 has been implicated in multiple signaling pathways, including BMP signaling, TGF-β signaling, Erk/MAPK signaling, Ihh signaling, and Wnt signaling, in the development of the skeletal system.^21^ Tang et al. reported that BMP signaling plays an extremely important role in osteogenesis and that Ihh signaling determines chondrocyte-to-osteoblast lineage commitment.^12,21^ In the current study, we examined the targets that interact with Runx1 by KEGG enrichment analysis and found that these signals were still dominant in chondrocytes (Fig. 5b). Among the targets that were shown to be directly related to cartilage pathology, three out of five were linked to the following pathways: BMP5, BMPR1B, and *FGF20*. BMP signaling has been reported to accelerate the differentiation of both chondrocytes and osteoblasts.^28^ BMP2 has been confirmed to increase hypertrophic chondrocyte differentiation^29^ and enhance the expression of genes, including Ihh, parathyroid hormone-related protein, collagen X, and osteocalcin.^30^ BMP7 has been shown to mediate cartilage metabolism and induce the synthesis of extracellular matrix.^31^ Recently, BMP5 has been reported to be highly expressed in articular cartilage in OA and could regulate chondrocyte senescence and apoptosis.^32^ The BMP type I receptor BMPR1B also plays a fundamental role in cartilage development and endochondral ossification.^33^ Our study provides a link between Runx1 and BMP5/BMPR1B in cartilage pathology. However, these two targets did not have motifs in their promoters that could directly bind with Runx1, but the other three (*TAPT1,* *RIC1*, and *FGF20*) were verified to have motifs in their promoter regions that could directly bind to Runx1 (Fig. 6d–g). *TAPT1* is a relatively new gene that has been reported to be involved in cartilage and bone development,^34,35^ and *RIC1* is associated with musculoskeletal and dental conditions.^36^ Their interaction with Runx1 might expand the understanding of the role of Runx1 in cartilage pathology. Fibroblast growth factors (FGFs) are important and relevant growth factors in cartilage development.^37^ Among them, FGF2 and FGF18 have been confirmed to participate in cartilage remodeling.^38^ However, FGF20 was also recently proven to regulate the proliferation of chondrocyte progenitors and promote mesenchymal condensation.^39^ This study also provides new clues for the roles of Runx1 and FGF20 in skeletal disease occurrence.

We previously used *Runx1*^*f/f*^*Twist2-Cre* and *Runx1*^*f/f*^*Col2a1-Cre* to elucidate the role of Runx1 in bone formation during different differentiation stages.^21^ These results showed the role of Runx1 in modulating cartilage formation, bone density determination, and osteoporotic phenotype generation. This study indicates that AAV-Runx1 overexpression protects against cartilage destruction in OA. AAV-Runx1 overexpression can rescue bone loss in osteoporosis by regulating osteoblast proliferation and differentiation and cooperate with Cbfβ.^21^ Runx1 overexpression in calvarial cells with conventional Runx2 knockout (*Runx2*^−/−^) rescued osteogenesis and mineralization, as shown by ALP and von Kossa staining, respectively, indicating that Runx1 could compensate for the loss of Runx2 expression. Taken together, these results suggest that Runx1 might be a potent therapeutic target in OA and osteoporosis.

## Methods and materials

### Runx1-knockout mice

The animal experimental protocol was approved by our Institutional Review Board (WCHSIRB-D-2017-029). Mice that carried floxed alleles (129-Runx1^tm3.1Spe^/J) and *Col2Cre* (*Col2CreER*^*T*^, tamoxifen-inducible) were obtained from Jackson Laboratory. The loxP sites were located on either side of exon 4 of the *Runx1* gene (murine *Runx1*, chromosome 16 C4, containing 8 exons in total). *Col2al1-CreER*^*T*^ was designed with the mouse procollagen type II, alpha 1, promoter sequence, *Cre-ER*^*T*^ fusion gene and *Col2a1* intron 1 enhancer. Transgenic mice with *Col2al*-*CreER*^T^ showed strong tamoxifen-inducible activity of Cre recombinase but had minimal activity in the absence of tamoxifen. These two types of mice were crossed to generate heterozygous mice (*Runx1*^*f/+*^*/Col2al-CreER*^*T*^), and homozygotes were finally obtained by heterozygote inbreeding. Tamoxifen was used to knock out Runx1 within 1 week of establishment of the OA model.

### Anterior cruciate ligament transection OA (ACLT-OA) model

We used ACLT surgery to establish an OA model as previously described.^24^ ACLT surgery was performed on the left knee joint of 8-week-old mice, while the right knee joint cavity was opened with surgical scissors and acted as the sham group. For AAV-Runx1 joint cavity injection, ACLT surgery was performed on both the left and right knee joints (left was the AAV-Runx1 group, and right as the AAV-GFP sham group). The mice were randomly chosen based on body weight and matched numbers of females/males. The mice (*n* = 15–20 in each group) were necropsied at 12 and 24 weeks after ACLT surgery. The knee joint samples underwent pretreatments according to the different experimental protocols.

### Hematoxylin & eosin (H&E) staining

H&E staining was performed by using hematoxylin (No. 03971, Sigma, St. Louis, MO) and eosin (HT110232, Sigma). Color separation was performed by using HCl-ETOH (1%, v/v) and ammonia-H_2_O (0.2%, v/v). Tissue section samples were then dehydrated with xylene and mounted with resin. Images were obtained by microscopy (BX53, Olympus, Japan).

### Masson trichome staining

Masson trichome staining was performed using a Trichrome kit (Trichrome TISSUE-TROL™ Control Slides, TTR012, Sigma). The experimental procedures strictly followed the instructions of the manufacturer. For knee joint staining, the cartilage stained purple, the mineralized area was green or blue, the steroid region was orange to red, and the nucleus was blue to gray. The tissue samples were dehydrated and mounted. Images were obtained by microscopy (BX53, Olympus, Japan).

### Safranin O staining

Safranin O staining was performed to examine proteoglycans in cartilage. Before safranin O staining, the tissue slices were pretreated with Weigert’s iron hematoxylin (HT1079, Sigma) for 5 min. After removal of Weigert’s iron hematoxylin, the tissue slices were washed with 1% acid-alcohol (v/v) three times. Safranin O solution (2%, w/v) was added and incubated for 30 min. Fast green (0.02%, w/v) was used as a background counterstain. The tissue samples were dehydrated (ethanol) and mounted. Images were obtained by microscopy (BX53, Olympus, Japan).

### In vivo imaging of the mouse model

An in vivo imaging system (Bruker In Vivo Xtreme II) was used to examine joint destruction in anesthetized living mice. Briefly, the mice were first anesthetized (chloral hydrate solution) and placed in the shooting box in an adjusted position. Images were obtained by radiography. This in vivo imaging system could provide us with a completely clear image of the joint cavity.

### Micro-CT (μ-CT) bone analysis

Micro-CT (mCT50, Scanco) was performed to examine changes in subchondral cancellous bone in older mice. The knee joints were pretreated with 4% PFA overnight, after which the PFA was washed away with water, and the samples were dehydrated in ethanol. The samples were loaded into scanning tubes and imaged. A Gaussian filter was set (σ, 0.8, and support, 1.0) for all image analyses. Other parameters included X-ray tube potential (55 kVp), X-ray intensity (145 mA), threshold (220 mg·cm^**−**3^), and integration time (200 ms).

### Chondrocyte isolation and culture

Chondrocytes were obtained from newborn mice.^40^ Chondrocytes were isolated by trypsin-collagenase digestion (0.25% trypsin for the first 30 min and 0.2% type II collagenase (Sigma, MO, USA) for 12 h at 37 °C). Chondrocytes were incubated at 37 °C in a 5% CO_2_ incubator. We used the first two passages of chondrocytes in the current study.

### Lentivirus transfection of Runx1 in chondrocytes

A mouse-runx1 overexpression lentiviral vector was constructed by Hanbio Biotechnology (Shanghai, China). The vector was based on pHBLV-CMV-MCS-3FLAG-EF1-ZsGreen-T2A-PURO. The M-runx1 overexpression vector was verified by sequencing. The virus packaging system as composed of pSPAX2, pMD2G, and shuttle plasmids. Transfection was performed by using a liposome transfection kit provided by Hanbio Biotechnology (1 × 10^5^/mL chondrocytes and 1 × 10^8^ TU·mL^**−**1^ virus).

### AAV-Runx1 animal model

Runx1 overexpression AAV was constructed by GENECHEM (Shanghai, China). The vector information was COL2A1p-MCS-EGFP-3Flag-SV40 PolyA. After the mice recovered from ACLT surgery, AAV-Runx1 was injected into the joint cavity (3–5 days post ACLT, 1 × 10^11^ v.g. per mL, 5 μL per joint). Knee samples were collected at 12 and 24 weeks after surgery to examine the effect of AAV-Runx1 overexpression.

### Quantitative real-time PCR (qPCR)

qPCR was performed to examine changes in target genes. Briefly, mRNA was isolated from chondrocytes by an RNeasy Plus Mini Kit (Qiagen, CA) and reverse transcribed to prepare first strand DNA by a cDNA synthesis kit (K1621-RevertAid, Mbi, MD). qPCR was carried out on an ABI 7500 system (Applied Biosystems, Shanghai, China). The cycle threshold (CT) was used to calculate the fold changes by the 2^−ΔΔCt^ method. GAPDH was used as an internal control gene. We used siRunx1 (mouse, sc-37678, Santa Cruz Biotechnology, Delaware Avenue, CA) and siRNA-A Control (sc-37007) to knock down Runx1, and lentivirus transfection of Runx1 was performed to overexpress Runx1. qPCR was performed to examine changes in genes induced by Runx1 knockdown or overexpression. Primer information is shown in Supplementary Information File II-4.

### Western blotting

Western blotting was performed to measure protein changes. In brief, the samples (tissue and cell samples) were lysed in RIPA buffer (P0013C, Beyotime, Guangzhou, China) and mixed with sample buffer (No. 1610737, Bio–Rad) at a 1:1 ratio. Equal amounts of protein samples were separated by 10% SDS–PAGE and blotted onto PVDF membranes after transmembrane electrophoresis. The antibodies used included Runx1 (No. 39000, ChIP grade, Active motif, Carlsbad, CA) and FLAG (ab125243, Abcam, Cambridge, UK and 9A3, Cell Signaling Technology, Boston, MA, 1:1 000 dilution). The secondary antibodies included mouse anti-rabbit IgG-HRP (sc-2357, Santa Cruz Biotech, Delaware Avenue, CA, 1:2 000 dilution) and m-IgGκ BP-HRP (sc-516102, Santa Cruz Biotech, 1:2 000 dilution).

### Immunohistochemistry

Immunohistochemistry was performed to examine the localization of collagen II (SAB4500366, Sigma), proliferating cell nuclear antigen (PCNA, 200947-2E1, ZenBio, China), TAPT1 (27657-1-AP, Proteintech Group, Chicago, IL), protein RIC1 homolog (RIC1, CSB-PA671121LA01HU, CUSABIO, China) and FGF20 (251634, ZenBio, China). After antigen repair for 10 min at 100 °C, the tissue slices were permeabilized with Triton X-100 (5%, v/v) for 5 min. After being washed with tap water three times, the slices were incubated in H_2_O_2_ (0.5%, v/v) for ~30 min and blocked with unrelated serum. The primary antibodies (1:400 dilutions) were then added and incubated for 2 h at 37 °C or overnight at 4 °C. The secondary antibodies included mouse anti-rabbit IgG-HRP for collagen II (1:1 000 dilution) and m-IgGκ BP-HRP for PCNA (1:1 000 dilution). Staining was performed by using a Vector^®^ M.O.M.™ Immunodetection Kit (PK-2200, Vector Laboratories, Burlingame, CA). The tissue samples were dehydrated (ethanol) and mounted. Images were obtained by microscopy (BX53, Olympus, Japan).

### Immunofluorescence analysis and confocal laser scanning microscopy (CLSM)

Immunofluorescence was performed to examine protein expression and distribution in the joint tissues. Tissue slice pretreatment was the same as that for immunohistochemistry. The primary antibodies included SOX9 (ab3697, Abcam, 1:200 dilution), Collagen X (ab58632, Abcam, 1:200 dilution), and MMP13 (ab39012, Abcam, 1:200 dilution). The secondary antibodies were anti-rabbit IgG H&L (Alexa Fluor^®^ 488, ab150073, and Alexa Fluor^®^ 647, ab150075, 1:200 dilution). 4',6-Diamidino-2-phenylindole (DAPI, D9542, Sigma) was used for nuclear staining. CLSM (FV3000, Olympus, Japan) was used to examine fluorescent staining.

### Chromatin immunoprecipitation sequencing (ChIP-seq) and ChIP-qPCR

For ChIP sequencing, chondrocytes were transfected with Lenti-3 × Flag-Runx1 (Lenti-3 × Flag-GFP as control). Coimmunoprecipitation was performed by using the Pierce^TM^ Agarose ChIP Kit (Lot#: TA265476). Runx1 overexpression was first examined by western blotting to confirm the success of transfection (Runx1 antibody and Flag antibody were both verified). The antibody used for ChIP sequencing was the FLAG antibody (ab125243, Abcam). After immunoprecipitation, the DNA fragments were sequenced and identified at Shanghai Lifegenes Biotechnology (Shanghai, China).

### Bioinformatics

Gene information was obtained from UniProt (https://www.UniProt.org/), promoter information was obtained from the National Center for Biotechnology Information (NCBI, https://www.ncbi.nlm.nih.gov/), and basal gene expression was obtained from BioGPS (http://biogps.org/). Promoter binding prediction was performed with PROMO (http://alggen.lsi.upc.es/cgi-bin/promo_v3/promo/promoinit.cgi?dirDB=TF_8.3). The online R language tool was used for KEGG pathway and GO analysis.

### Statistical analysis

Data in the current study are presented as the mean ± SD (detailed in the source data). All statistical analyses were based on two-detailed Student’s *t*-tests (small sample statistics). In each analysis, the statistical threshold was set as 0.05.

## Supplementary information

Source Data

Supplementary figures

Supplementary Information file-II

Supplementary TableS1

## Data Availability

Raw data are mainly attached in the current study. Any other data involving this study are available from the corresponding author upon request.
