# Supplementary material for: Runx1 protects against the pathological progression of osteoarthritis
Source: Bone Res. 2021 Dec 7;9:50. doi: 10.1038/s41413-021-00173-x (PMC8651727; doi:10.1038/s41413-021-00173-x)
Supplement: Supplementary file 2 — Supplementary figures [file 41413_2021_173_MOESM2_ESM.doc]

*Original article*

**Runx1 protects against pathological progression of osteoarthritis**

**Supplementary figures**

**FigureS1. Establishment of Runx1 knockout in articular cartilage in Runx1*^ff^*Col2-CreER mice by tamoxifen.**

**a.** Immunofluorescent staining showing Runx1 knockout in hyaline cartilage of the knee in Runx1*^ff^*Col2-CreER mice. The samples were collected at 3 days after first injection of tamoxifen. Col2a1 (green) and nuclear (Dapi, blue) were counterstained. The images were chosen based on three independent experiments (n=3).

**b.** Immunofluorescent staining showing Runx1 knockout in growth plate cartilage of the knee in Runx1*^ff^*Col2-CreER mice. The samples were collected at 3 days after first injection of tamoxifen. Col2a1 (green) and nuclear (Dapi, blue) were counterstained. The images were chosen based on three independent experiments (n=3).

**FigureS2. Establishment of Runx1 overexpression in articular cartilage by adeno associated virus (AAV).**

Frozen section showing success of AAV-Runx1 overexpression in articular cartilage of knee joint. The samples were collected at 12 weeks after AAV injection. Nuclear (Dapi, blue) were counterstained. The images were chosen based on three independent experiments (n=3).

**FigureS3. All Homer *de novo* Motif Results pull down by Flag tag (3× Flag-Runx1-GFP)**

**a.** All motifs by homer *de novo* analysis pull down by Flag tag.

**b.** Detailed motif information about TTTTGTAGAA.

**c.** Detailed motif information about TGACTCAC.

**Figure S1**


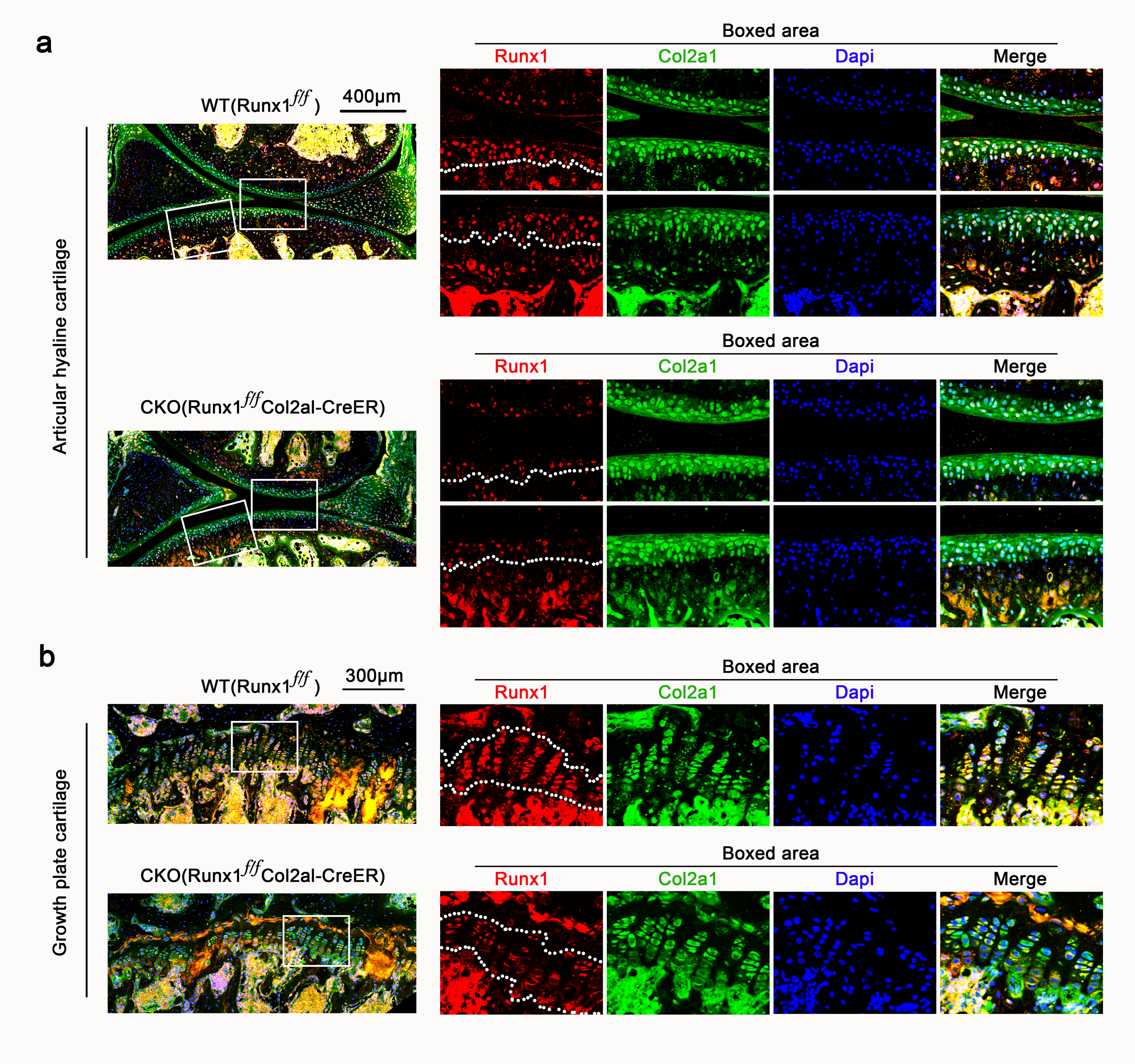


**FigureS1. Establishment of Runx1 knockout in articular cartilage in Runx1*^ff^*Col2-CreER mice by tamoxifen. a.** Immunofluorescent staining showing Runx1 knockout in hyaline cartilage of the knee in Runx1*^ff^*Col2-CreER mice. The samples were collected at 3 days after first injection of tamoxifen. Col2a1 (green) and nuclear (Dapi, blue) were counterstained. The images were chosen based on three independent experiments (n=3). **b.** Immunofluorescent staining showing Runx1 knockout in growth plate cartilage of the knee in Runx1*^ff^*Col2-CreER mice. The samples were collected at 3 days after first injection of tamoxifen. Col2a1 (green) and nuclear (Dapi, blue) were counterstained. The images were chosen based on three independent experiments (n=3).

**Figure S2**


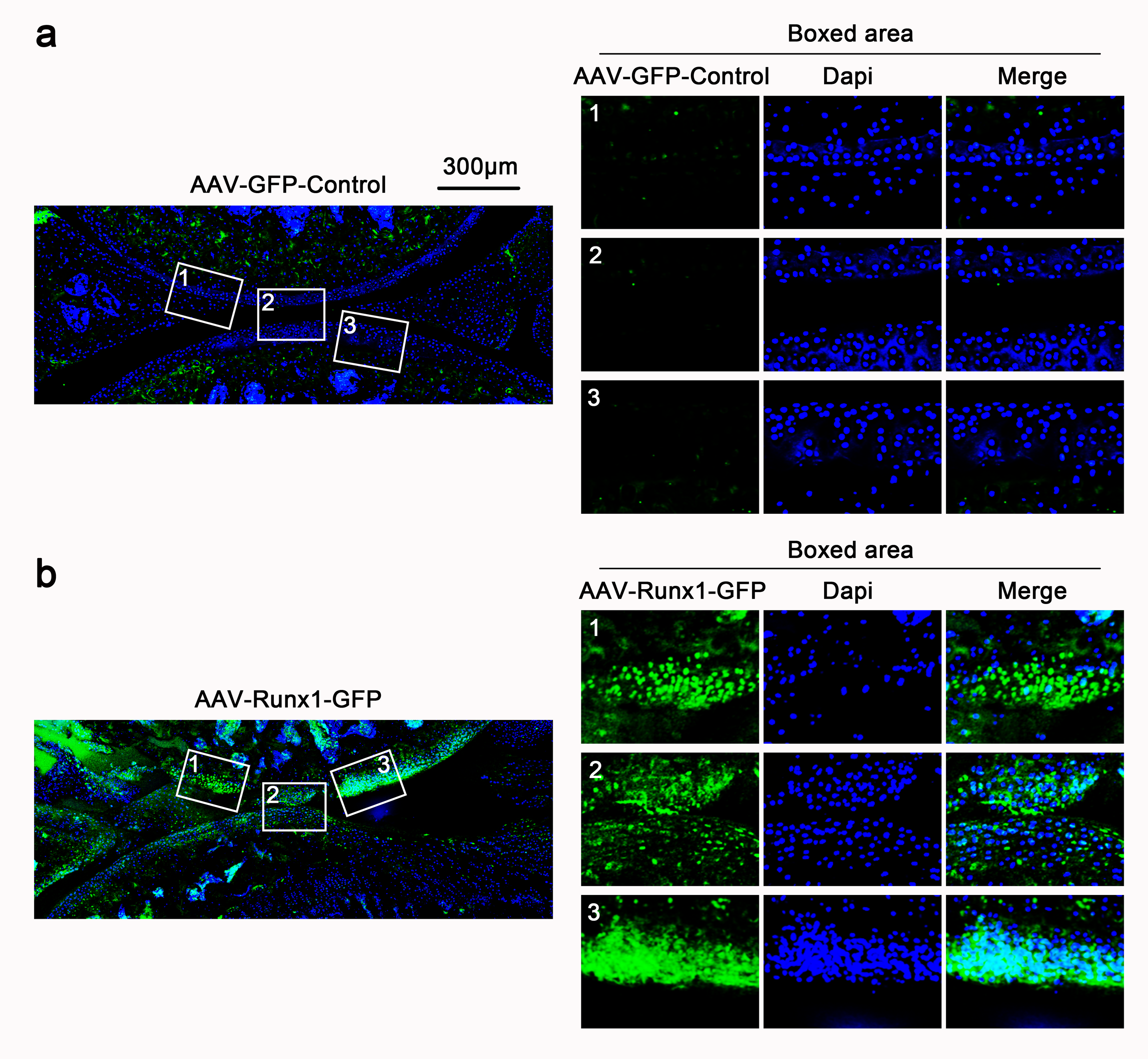


**FigureS2. Establishment of Runx1 overexpression in articular cartilage by adeno associated virus (AAV).** Frozen section showing success of AAV-Runx1 overexpression in articular cartilage of knee joint. The samples were collected at 12 weeks after AAV injection. Nuclear (Dapi, blue) were counterstained. The images were chosen based on three independent experiments (n=3).

**Figure S3**


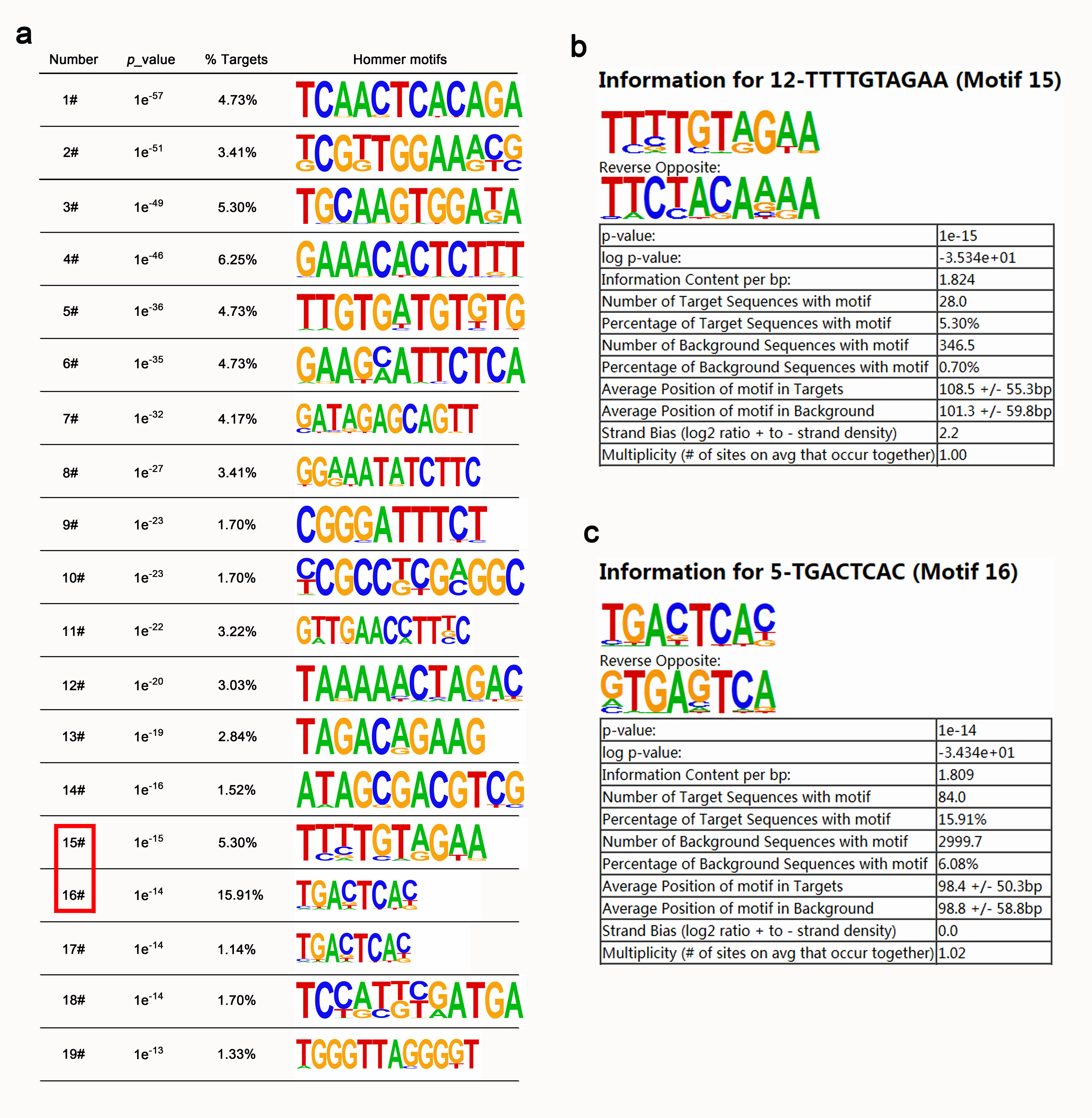


**FigureS3. All Homer *de novo* Motif Results pull down by Flag tag (3× Flag-Runx1-GFP). a.** All motifs by homer *de novo* analysis pull down by Flag tag. **b.** Detailed motif information about TTTTGTAGAA. **c.** Detailed motif information about TGACTCAC.
