# Supplementary material for: Runx1 protects against the pathological progression of osteoarthritis
Source: Bone Res. 2021 Dec 7;9:50. doi: 10.1038/s41413-021-00173-x (PMC8651727; doi:10.1038/s41413-021-00173-x)
Supplement: Supplementary file 3 — Supplementary Information file-II [file 41413_2021_173_MOESM3_ESM.doc]

*Original article*

**Runx1 protects against pathological progression of osteoarthritis**

Supplementary information about detailed promoter binding sites and primer design

**1. Tapt1 transmembrane anterior posterior transformation 1 [ Mus musculus (house mouse) ]**

The detailed information about Runx1 at the promoter of ***Tapt1***:

The following sequences in capitals at the promoter of ***Tapt1*** (Gene ID: 231225, Chromosome 5 - NC_000071.7, **-4000** bp in capitals ~ **+200** bp in grey (Transcriptional starting site, TSS))

**Runx1: primer design for TCTTGTAGAA**

Candidate primers:

Primer pair 1# (125bp): Forward: TGGATGTCCCCAGCAGTTTC

Reverse: CCACACAGGAGACAGAGCAG

Primer pair 2# (122bp): Forward: GATGTCCCCAGCAGTTTCCA

Reverse: CACACAGGAGACAGAGCAGG

Primer pair 3# (137bp): Forward: CTGGTCCTTAGGCTGGTGTG

Reverse: AGGAGACAGAGCAGGACCTT

**Runx1: primer design for TGACTCAC**

Candidate primers:

Primer pair 1# (148bp): Forward: TAACGGCCCTTCCTGTTTCC

Reverse: TCAGCCTCTGATAGGCAGGT

Primer pair 2# (142bp): Forward: GAGTAACGGCCCTTCCTGTT

Reverse: GATAGGCAGGTCTGTGGCAA

ACTCATATGCCTTTTCCTCTTAGTTTTAGCTCTCTGATTGTTAGGTAAGCAGCCTTGCTTTGCCTCATGTTCCTGCCATCAGGAGCTGCTTCACTACAGGCCCAAAGCAATAGGGAAAATTGACCATGGATTGGGATATGTAAACTTGAAGCTAAGAAAACCTTTGCATTCTGGACTGAGGTATCCTCTGTAAAAGTAGCCAGCTGAGCACTCTGGGTGGAACCTGAACTGCTTTGGTTTTATTTCATCTTACCAAAGCTTTAGCAACCCAGCCTTCTGCTCAGCTCATTCTGGTGTTCAGCATGGGGTTCACAGACCATAGTCTTTCTTTGGTCTCATAGAAATGTTTGTCCTCCACTATGGCCCGTGAGCAAGGCCAGCTTGACCCTGGAGTCACTCCTGGAGCACGGCAGATTTGAAGAGGGTTCATCATGTGCTCGACAATGAGAAACAAAATCAGATAAAAGTTTGGCTTTCCACATGGTGTCAGCGCTGAAGTAAATGGACCAAGTCTGGGGTCAGGATCCTCTTTTTGGAGTTCTCAAGGGATATTGGAAGGCACAGGACTTCAGAGCAGTCTGGAGTCGACACTGCCATAGTATGGCTCCAGGTCTGCATGAGCCCATTGCATTCCATGCAGACAACCCACTAGCCTCCTTGATCCCTTGGCACTGCACTGGGTACCCACACCAACAGTATCTTAGCTTCTCTGGTCCTTAGGCTGGTGTGGATGTCCCCAGCAGTTTCCATACAGGGCCTCCTTTTCCCAACCCTTC**TCTTGTAGAA**CATAGCTGATGGCACAGGGCAAGACTGTAGAAAGGGGTTCAAGGTCCTGCTCTGTCTCCTGTGTGGTGGGCACACATTACTCTTCCTTTCCAGATTGCGTTCCTAGCTGTAGGATGGGGACGACAGCATGCACTCCGTAAGGGGATGAAGATGTAGCAGCTGAGAACCACACTGTGCCTTCACACAAACCTCCAACAGCTATCAGGTATCAGTCCTTTGGGAGGTCAGCAGCCTGCCTTTCTTAACACATTGACCTAATCATAGTACTTGAAAGTGTCCAAAGTAGCTTCTTCACTTATCCTCTGTATACGTGAACTTAACATCCCACAGGAGCTCGCCTCTGTCCAGCCTCTGAGCGGAATCAGGGGCAGAATCCTCAGCCAGGCACTAAGAGCTCTCAAGCCCTGTCTCCATATAGAGTCCCTGCCACCTCCAACCAGAACTGCCTTCCTCTCTGTGGTTTCAGTAGTGGCTGGCTCTCAACACACATACACATACACACACACACACACACACACACACACACACACACACACACAGCGTGTGTTTATGTGTGTATAGGGGCCAAGGCTGTGCTGTAGACATACTGTGTGCTCTTCCCTCTTCAGTGACAACTACCTTGCCCTTCCTCCCCTTACCCTCAGCCCTAATCTAACAAGGCATCTTAAATTTAAGTGCACGTCCCTCACAGTGGCTCTTTTTAAATTCTTGCAAGCAGTACTGTTGGTCTCTATCAGGAGCTGAGGGACAAGGGATGCAGCACCATCTAAGGTGGTCTCCTGTGATCAGTGTCTGCAGACAGGGACAAGAGTGTCTCGTTGTCAGGTGTGCATTTAGCTGCCACTTCCCTTACCCACTAGACAAGATCCACTCACAACCACACAGCAGCACTTGTTCATTTTCGTGCCTCAAAGTAACTGGGACAGTAATGCCTACGAGCTTTCCTGTCTGCATTGCACTGGCCTTGTTAACTCGCTAGAACAGCATCTCTGACTTTCCCAGTATGTACGTTCCTGTGATGACAGGACCCTCATGAACTCCTTGGTTTCCTCATTGCTAGATGAGGTTGTATGTTCAAAGCAAAACTCTACCAACTGATGCTCACTCTTCTCTTCCGGTAACGACATCTCAAGAGCAGCCTCACCGGCCGGCAGAAGACCCCATCCCTCACTACCTTGCTCATGTTTAATCAGTCATGTCTTGTTTGTTCACCAGCCCCTAGGAACTCACCGTTCCCCATCCTTGAAACAAACATTTGAGAATCACTGAAACCTTCTCACCATGCCGATTTCTCCTGAGTAACTCACACCTCCCTCATCACTGACACTCCTTCCCTCACTAGGTGTTTTACAAGAAAACACTCTAAATCAGTGGGTCTCAACCTGTGGGGCCCAACCCCTTTGGAGGGGGGGGGGTCAAGTGACCTTTTCACGGGGGTCACCTAAGACCACTGGAAAACATAGCTATTTATATTACAATTCAGAACACTAGCAAAAATTATAGTTCTGAAGTAGCAACAAAAATAATTTTATGGTTGTAGTTACCACAACATGAAGAACTGAATTAAACTGTATAAAGGGTTGTAACATTTTGAAGGCTGAGAACCACTGCTCTAAAACACCCTGGATGTCTGAGGCATTTAATGAATACCACATACCACACTGAACTGTACCACAGGACTATTTTCGACAACCACTGGCATGGCATCCTTGGCTTCTGAGATATTTTTTTACCGCTTTCTTGGCTCATTAACATTCTACTTCTTGCTGTGCCCTGCGCACCCCCTCCCCCCCCCCCCCCCCCCCGTGTGTGTGTGTGTGTGTGTGTATGTGTGTTTGTAGGTCAGCGATCAACTTGTGTGAGTTGTTTCACTTCTTCCACAGTGTGGAGTCTGGGGATTGAATTCATATTGTCAGGCTTCGCAGCAAGTGCGTTTACCCGCTGAGCCATCTTGCTGGCTCCTTATATGTAAACTCTTACACATTCTTTCCTGACATAAAGGGCAAGAAATACCTGCTCACTAAGTCTGTGAACAAATCAGACTTTAATGTTCATCTCCAGGGCATGAGGGGTGTCAGCTCTGAAATACAGGGAGTAACGGCCCTTCCTGTTTCCTCTAC**TGACTCAC**GGTAAACAGGGACTCTACTGTCCACAGGGAGTGTTGCCGCAGCCGGAGTATCAGAGGGGTCTGACGCTCATCTCCTTTTCCTAATGTTGCCACAGACCTGCCTATCAGAGGCTGAGAACACAGGTGTTTCCGAATTTCTTTCTCTTTCATTAGAGTCTTGAGTATTCTAAGCCAATCAGCCATATTCCTTTTCACAGGCATCCTGGGCCTCAGGACCTTGACTCTGCTACTCTGGGGTGCTGTGCAATATACATGGCTAGGGATAGCTACCAGCTTCTCGGGGTTCCAAGGGTGCGAAGTGGACAGACTCGAAGGTGACTTACAGTATGATGGGCATAGACTTGAAAATGGCATATTAACCTCTAGACTCACAGTGAGAGGCCATGAAGCTTGGCTGCAGTCCTGTACACAGCATGCAGGAAGGCCAGAGTCTGGCAGGCAACTAGCAGTATTTGGTGAAAATTCTTTTCCTCTCTCTTCACCCCTTTTTAGAAAGCTCTGGGAAACTGCTACCAGATGACCTTAAGCACACAGCACAGGGAAAGATCAGCCTGACACCACGACGTGAACTGTGCTATCCTTGGTCTGCCATACATACTCGACCAATGTCCAGTTGTGTCCAGCAGGCAGAGACCTTGCTGTCTCCTGCTATCCCTTGCTCTGGGCAGTTCCTGACAGAGGAAACAACTAAGAAATCACTGTGGGAAAAACACTTAGAGCTGAGGCATATGAAAATAAAGTACTTTAAGGCAATCTTGTGAACTCATCTCTGCTGAGCTAGCAGAGGTGGCAGGGCAGGTGGCACAGAGTGGGTGAGGCAGATGCTGAGAAGCTGATTCAAGCATACTTTTTTCAAATGCTGGCACAAGACCACAGGGAGCCAAGATACATTCATCAATAGCATTTGGTACTTTGACCTGTAGGAAAAGCTAAAATGGGTCTGATGTACCGTGCACCATACTAAGACTATTTAAAAACCAAAGTATTGTCTTGACTGTGACTCCCATATTATGACAAGAAGACACTGTGACATCAAGATGCACCATAGAATGCAAATGTTGTCATGTGATAAGAGATACTAAATGGTCTATGACCAACATGCAGGCTATTCTTTCAAAGACAAAAGAGCAATTTTTTTTTTATCATTTAGGGCAAGAGTAACTAAACATACTTAACACAACCCTATTATTTGTAAACTAGAAATAAGTTCTGCTTAAATAAGAAATCTCTCAATGAGATATCCATTCCAACAAATATAATAGTAATCGTTGCCACAAGAAATTCAG

**2.Ric1 RAB6A GEF complex partner 1 [ Mus musculus (house mouse) ]**

The detailed information about Runx1 at the promoter of ***Ric1***:

The following sequences in capitals at the promoter of ***Ric1*** (Gene ID: 226089, Chromosome 19 - NC_000085.7, **-4000** bp in capitals ~ **+200** bp in grey (Transcriptional starting site, TSS))

**Runx1: primer design for TGACTCAT**

Candidate primers:

Primer pair 1# (94bp): Forward: TCTCTTGTTGAGCACTCCTGG

Reverse: ACAGCCCCATCATGCTCTATG

Primer pair 2# (192bp): Forward: ACTCATAGAGCATGATGGGGC

Reverse: AGGCCCTGGCTCAAAGAATG

Primer pair 2# (190bp): Forward: CATAGAGCATGATGGGGCTGT

Reverse: GAGGCCCTGGCTCAAAGAAT

AAGAAAGTTCCAGGAGTAAAATACTCTGTGAGATCACTGGCAAAGTGGTGGAGTTTTATAAACAACTGGGGAACATTTTCCCAGTGTTTAGTTGGGTTTCGTTTACTTTCCCTTCTTGACTTTTTTGAACAACCAGCAGACTTATTTTGATATAAAAATTCAAATTCCTTTGTTCTGTGGCCATTGTGATTAAAGATAAAATCGTCGCTGCTCAGTTCCATTCTGGAACGGAGGGTAGCCGAAGCTCCAGCATCTTAGGATGCTGCTGGCTCTCTGCACTGTCAGTGTGAGGCGTGTAGTCACGTGCGTGCACACCTAATGTCTGTAAGGAAAGGAAAGGGATTGGGGGGGAGTTACATGCTTTAATTTGAAATAAAGTCAGAATTAACTTTTTTCCCTTAGTTTATCTCTGGGCCTTAAATTTTTCTTCAGTTGGTGTACTAGTTTTGTGTGGCTAGTTTTGTGTCAACTTGACACAGCTGGAGTTATCACAGAGAAAGGAGCTTCAGTTGAGGAAATGCCTCCATGAGATCCAGCTGTAAGGCATTTTCTCAATTAGTGACCAAGGGGGAAGGTCCCCTTGTGGGTGGTGCCATCTCTGGGCTGGCATTCTTGGGTTCTATAAGAGAGCAGGCTGAGCAAGCCAGGAGAGGCAAGCCAGTAAAGAACATCCCTCCATGGCCTCTGCATCAGCTCCTGCTTCCTGACCTGCTTGTGTTCCTGTCCTGACTTCCTTTGGTGATGAACAGCAATGTGGAATTGTAAGCTGAATAAACCCTTTCCTCCCCAACTTGCTTCTTGGTCATGATGTTTGTGCAGGAATAGAAACTCTGACTAAGACAGTTGGATTAAAAAAGTATAACATTGAATTAAAGTGGGTAACAATGTCAAAAACATCGATCTGCTGTAGTAAATAATATTTGGTGGTTTCTATCTTACCTTAGATCATTTAGTTTCCAAATTAAAGACACAAAACCTTTATGTTTAGAATAAACCTTAAAGCATTAGAAGTGGGCAGAAACTCCTCTAAGCTAGTGTGTCCACTTCCCTGTCAAGCCTGAGCGTCACTTGCCATATTCCGCCTGGGCCACTCCTACTGCACCGTGACAGTCCTCATGGCCGTGCTCTCACCTACACTGTGGTGGCTGCTTCCTGCTGCTCTGTGCTCCTCACATCCCTCCTCTTTTCATGGCCCCTGTCTGGAACCCCCGAGCCTGGAACCCCCGAGCCTGGGGTCTTGAAGTTCTGCCTATCTCTCTTCTGCCCAGTTATCTGTAGGCATCTTTATTAACCAATTGTGGGTAACTTACAGGGCAAGTTTTATACAATAAAAGCTGTATATGTGAGGATCTGCTCATCTTGAAGCAACTAGATCTTAGAAAATTAAGCATTTGAATATGTAGCAGCACATTGATCTTTTAAACAAAAATTTGTAATTAGAGGCCACACACATCCTAAGATCAATACTGATAACTGAAAATATTTTATATAATGGAAACTTATAAATTATATTACATTGAAATATTAGTATTAAATTATTTAACTTATTGCAAGTTTTGTAGGTGTAAGAGAAGTTGAGACATATTCATTCTCTTGTTGAGCACTCCTGGAGTCCCTGTATATTACAGGTAGCATGTAATCAATGGAGAAGTTTTCT**TGACTCAT**AGAGCATGATGGGGCTGTACAGAGCATGTGTGGAGGGCAAAGAACAAATTACAGGAGTCTGTGTTCTTCCACCATGGTGGTCCTAGGGATCCAATTCAGGTTGTTAGGTTTGGCAGCATGTATGTACCTTTAACCAGTGAGTGCTCTTGCTGGCTTAGAATACATTCATTCTTTGAGCCAGGGCCTCAGAACCTTCCATGTAAACTAGGTTGGTCTCAACACACAGAGATCTGCTTGATTCTGTCTCCCCATTGTTGATTAGAGCTGTGTGTCACCATGCCTAGCTAGGAAGAATAATTTTTGATAAATGCTGATCAAATTAAAGGACGTGCATCACCTTGTTTCAGCATGTTTGTTATCCTACCATGTTCCATTTGTGTTTATTGATGTCTCTATGTCCAGTAGGTCCGTTGCTATCTTGGGAGACTGGTGTCATTTGTGATCTACATGCTTTCTTATTCCTAACATGTATAATAAAGACCTTGAAAGTGAAAATGGAATTTACATTGCAGTCTGCTATTTCATAGTTATTGAAGAGAAATTATTAAATTGTGAATGGTTATATATATAACATATGTTTGTTATCAGAGGGAACTAAGATTGATACATGAAAAGGCTAAGCAATGGTTACATGCTTTCTTGATATCATTTGGGAGTCCTGGAGAAAGGTCATTATAATAGTGCATAGTAACAGATTTGGTGACTATTAAAGAGACATCATTTATTACTAAGACTTTAACAATTGTTCAAAGGCAGAACTGTTTGTGAAATGTCAGTAGTAGATGCCATTGAGATTTAAAAGTTTACAATGTTTGTTATACACAAAAGTTTGAAATATTTATTTAAAATGATTGATACCAAATATCAAATAGTAAATCAATATATTATTTGTGTTTTCAGTATTGTATTAGATGAAATGCTAGTACAAAGTGATGCTTTATTCTCATTGTCATTATAGTCCTGGGTATGCTTTATTATTTTGCTGTGTATTTTATTAGCATTTGAGTGTCTGAAGTGTTGAACATTTTGACCTTCGAGTGACTGATCTTCCCGACAGTATTTATGTTAATGTTACAATCTTAAGAGCTGACCATTCTTTTTGCCTTTCCTGCACTTATGAGGTGGCAGTAAAAGTTTACAAGTTGTGATTCTGTGTGGGGTAGGAAGTATTTATGACCTGTGCTGTGGTTTGATTTGAAGTCATTAGCCTTTTGATTTTCTATTGCAAGAATGTAAAATTGTCATGGAGAATTTGAAAGATAATCTTTCCTGAACAATCTTTATTTTCTTGTTTATATCCTAGTAAGTTAAACTAGTTTTTGAAATGATATTACAGAAGAATAAGCTATAGTTACCCATGGCTGCCCTGGGAAGTCTGTTTCCTTTCATTCCATAACAGAAAAGCAGGAAGCTAGCGCTGGGAAGAAATAGGAAGTCCTGGGTGATTTCTTTGGTTTGGATCATTTCTACTTTGGATACATCTTCACTTGCCTCTGAGTTAATAGAATTCAGTATTTGGGTAAAGAAGATTAGAGGGAAAACTGCCACTTCACTCTATTGCTTGATTTTATACGGAAAGTGTCTTCTAAGAGCAGCAATTCATAGTATTTAACATGTTTATGTAAATGTAATACAACAATGTTTTTGTATTGTTTGTGTTTTTGTTTTTTAGTTTTATTTTTGAGCTAGAATCTCTTAGGGTAGCCTAGGACAGGTAGAAACCTGCTTCAGTTACTCAAAACTACAATTTTTTAAAATTTAATTTCTATTTTTATAACATTTAGTTACTTTAGAAATTTAGATGTTTATATTTGAACTTCAAATGTTGGAAATGTTCAAATCTTGGAAATTAGAGATTGACTTAGCTGATGCTTATATTTTAAATTTTCAAGGTTTGCTCTTATCATTTTTCCTAGTGTCTCACAGTATGTTTTATAGTAAGAGGTTAAATTTTTTTATTTTTAACTATATAAATTATGCTGTTTTGATAAATGATAGTTTTAGGGGTACAGTCAGGCATTTTGTGCTTTTACTGTTTGGTTATATTGCTTTTCGAACTTAGAATAATTATTTGCCACACTTTTGTAGAGTTGAAAGAGTGCTGTGTATTTTACATAGTTTCATAAAATATTTCAATTTTCAGTTACATAGTTTATAAAATATTTTTAAAATTTGTTGAACACTTTTTTAAATGGCTTATATTAAACATCATTTTGTGTATATAAAGAGACTATAATTCTCCAGAGGAGGTATTGTAGTCCTGTAAAATTAGAGCCCAAGATGATAGTTACAACATTCTAATTCATGGTTAATAAACTTCTCTTTCAGACAGCAAATGGCTACATCTTGTTTTTTCATATTACATCTTCAAGAGGGGATAAATACCTTTATGAACCAGTGTATCCCAAGTAAGTTTATTCACTTTTATTCTTTTAATGCATGGCTTGCATGCTTAGTATTTTTTAAAAAATTGTACTTTGGGTTATCTAAAGTCATTAAGATGTTAAGTTGCCAACATTTCTAAAATGGTGCTTC

**3. Fgf20 fibroblast growth factor 20 [ Mus musculus (house mouse) ]**

The detailed information about Runx1 at the promoter of ***FGF20***:

The following sequences in capitals at the promoter of ***FGF20*** (Gene ID: 226089, Chromosome - NC_000074.7, **-4000** bp in capitals ~ **+200** bp in grey (Transcriptional starting site, TSS))

**Runx1: primer design for TCCTCTAGAA**

Candidate primers:

Primer pair 1# (239bp): Forward: TGATCACGTGGTGAAAGAAAACG

Reverse: GGCTTGAAAGGGAATTCGACA

Primer pair 2# (239bp): Forward: GATCACGTGGTGAAAGAAAACG

Reverse: AGGCTTGAAAGGGAATTCGACA

CAGAAAATAACTTGAAGAAGGAGCAATATAACAGAAATGCCACATGAGATTCCTCTACACACCCACTCACACCTTTAAGATGCTTATGTTTAAAAATGGAGGATGCTCACATCTGTGATTGACAGGGGAGCCCACAGTTCATTCTGGGAGATGCCATCCCTGAACAGCTGGTCCTTGGGTAGATAAGAAAGCAAACTGAACAAGCTAGGAGTAAACAATCCAGTAAGCAGCACTCCCCCAAGACTTTTGTGTTAGTTTCGGCCTCCAGGTTCCTGCCTTGAGCTTCTGCCCTGGCTTCTCTGGACAGACTACAAACTATAAGCTGAAATAAACTTTTCTGCCCAAGTTGCTTTGGGTCATAGTATTTTATCATGGCAATAGAATCCCAAGACAGTTAGAAAGTCGTGGGTTAGAAAGGCAGTAAGTGTGGCTTACTGTCATCAGTATTCATAATTTCTTGTACAGAATAAGCAGTTGGGTGACTTCATTCAGGTATAGAAATGTTTAGAAACAGCCGGGCATGGTGGCACATGCCTTTAATCCCAGCACTTGGGTGGCAGAGGCAGGATTTCTGATTTCTGAGGCCAGCCTGGTCTACAGAGTGAATTCCAGGACAGCCAGGGTTACACAGAGAATCCCTGTCTCAAAAAAAGGAAGAAAGAAAGAAAGAAAGAAAGAAAGAAAGAAAGAAAGAAAGAAAGAAAGAAAGAAAGAAAGAAAGAAAGAAATGTTTAGAAATGCACTCATCTAACTTTTAAGGCTAAGATCATATGCAAGGATACCCACCTCTATACTCACAGTTTATGATTTTTAAAAAATGAACACGAGAAAGTATTTGAGAAATCTTATCAATAATAAATGTGCCTTTAGTGAATGTATTAGTTACCTGTTCTATATGTTATAAGACTATGAAATCAGTGGTTTAAAAACAGAATGTGTTATTATTCTGCACTTTCTGTGGGTCAAGCAGTCTATCAGGATTTAAGTGGACCCTCAGCTCAGGGTAGAAGAGTTACAATTTGCAGGTTGCATTCTTGTCTTGAGGCTTGATTGGTTACAATTCACTGAGCTTTTAGTTATTGGCAGATGTTGGTTTCTTGCAGTTGTAGGACTGGGCGTGGCTTTGAACTAGTTATCAACTGGATGCTATTCTTGGCTCCTATAGGCCACCTGAGGCTTCCTGGTACTCTGGCTTTCCCCATCCCGGTGCTTACTTCATTCATTCAGCGAGGAGATTGTCTACTGCTAGCAAATGTACTACAGAAACAGTTTTATGTTGTCTAATTGTCACAGTGTCCCTCACTTTTCTATATTCTATTGCAAGGTAGAAGCTAGCCTGGTCTAATGAGTGAGTTCCCTGGCCAGATAGAGCTACATACAACATCCATTCTTTAAAGAACAAAGGAGGAAGAAAAGGAAATGGAAGATGCTCAAATCTCACCAAATAACCATTAAAAGTTCTTGTTTGGGTTTCGAATCCCAAGCCTAAGTCTCCACAGCCATCTCTGGCAACCCTCCTTTACCTGGTATTAGCTTCGCCTCTGCTTTGGGTTTTCCTTTATATCAGCTCTGAGAACTAGAATCTGGAAGCTTCCCTGTTTTTTTGTTTTGTTTTGTTTTGTTTTGTTTTTTTAAATACCTGCCATGTCAGGAGAAAACCCAGGAAGTCACATTAAAGCCCCAGAAGGGCTTTGTTTGATGTAGTTGGTTGATCCTTTCATTAGATTCTGTGACCAAGATGGTATGTATGATTAATCAACCTTGGACAACTAGCTCTAGAACTAAGTTTAGGATCAATCTGACTTCAACTAGATAAGTGTGAAAGCTTACTATGCATGCTAAAATGTTTAATTATGTAACGAAGAAAATGCTATGGCACTGTATGATGAAGGAGAGATTTTCAGATGGAGGTTCAAGCAACCAAAATACTATTCTAGTTTCTGATGCACCCCCTCCATGCTAACTCCTTTATTCATAAGAATAAAGCACTTGTATTCATTTCCACATCTGCAAAGAATAGATGTCATACCACCAAGTTCCTGAAAATAAGACAAACCAGCTCTACCCAGCATCCCTATTAGAGAAGAAGTCAATGCAGGGAATTGATTTGATGAAAATAAACCTGCTACTCAGCCAGATAGTGAGTTTTAGATATTCATGATACGAAAACAAGACCCCTGAAGCATGAGCCCGGAGGGAAGACTGTAACATCCCACTACTCAACAGCATGAGATGTGGGAAGAACCAGCAAATAAAGAAAGCTCAGAATCCCAGAGAAGGCAGAAACCATTCCCTCAGCTGCATTGTAGAGATTCCATTCCCATCAGAAGCTCGTCCGCTGATTAGGACTCAAGTTTATCACCAAGCTAACATGTGATCAGTGAGTTCAAGGCCAGCCTAGTCTACATAGTAAGCTCTAAATTAGCTAGAGCCACGTAGGCACTGTCTCAAAAGAACCAAAAGATAAAAGAAAAACATGGTGCTTCCCTTACAGAAATCTGTGTGAACAGGTGAGAAGGCAAATGGTCATTCATTCCATACAGATAAAAGTATCTTTGGGATTAAAACATTCCTGAAAAACCTCAACTTATAATTGTCTGGCCTTCAACATCTTTGACATCACCAATGGGACGTCAGGGAGTTTAGATTTCTCGAATGGAAGAGCCTTCATTGATTGTTTGCCTTGTTTACACATTTTGGCCTAGTCAGCTCTTAGCTCCTTCTTGTTGTTTTGAGTCTACCAGAAATTTCACCTCTTTAGAAAAGCTTTCTCTGGACCATCCACCCTCTGAGCCTGTTCTCTCTCCCTCTCCCTCTCCCTCTCCTCCCTCTCCCTCTCCCTCTCCCTCTCCCTCTCCCTCTCCCTCTCCCTCTCCCTCTCCCTCTCCCTCTCCCTCTCAGTGGCTAAAACTTGTTCGGCTGTAGAAGTACATTGCAGTTCACACTTAGAGTGTTTGTTAATTTTTGAGCCCCAGGATGCAGGGATGACTTCCTCTTGGGCATCACACCAATGCATTCTTTGGTACTCTCCACAATGTCTGCTGCGTAGGAATCATTAATGATATTTAATGTAGCCATGAGAAGAACACCAAATTCAGGTAGCTACAACTTTACCATTGTTAATGGAAGTTCTTTCTATGATCACGTGGTGAAAGAAAACGTTTTTCCCCTATATGAAACTAATAAAATAAGTAAACATAAATTGACTATATTATTTGATAACTTCAACATTGTATGTTCTAACTACTAAAGATTTTCCCCAGGGGACTTAGACTTTTCTCATTTTAAAATTG**TCCTCTAGAA**ATAGAACAGCTCTCACCTGAAAAATCAATGTTATCAAAAACATAAAATGAAAATGTCGAATTCCCTTTCAAGCCTGAGTTAAGTTCAACACAAAGCAATAAAACATTTTTCAAGGCCAAAAGGGACCCATGCTACTTTAGAAGACAAGTGAATGCATAAAACTATAATCTATGCTGTACTTAATTTTTACAAACAAATGAACATTTTATTCTGTGGCTAATAAATTATATAATAATGAAGGCTTCTTTATTTATTCCAAGACTAACACAATGCAATGGCACAAATAAAGGACACTTTGTTGCTCTTTTTGTTTGTTTTATTTTGTATAGTCTTCATATACTAGTAAAAATAAACTCTTTAATAAATAAATATATATAATAAATATTTGTAAATTGAAAAAAGTATAAATAGGTCCATTCTTCTAAAAGAAAAGTCCCATCTCAGTGTGGTGTGGTTTGTAGATCAAAGTCATCTAGTATTTTGATTTTTGTTTTATGTATAACGTTCTAACCTTTCTACAAGGTCCTCAGAATTTATGAGGCAGTAATTATGTCATCTCAATCCACTTCTTCAACATGAAGTATCTATTTTTAAGATAACTGTCCAAGTAAACATTATCCAGAGACATGCGGCGTCAGCAGCAAGGTTCACATTATCCGCAGATGAACTGATTTCAGCATCGTCAGTCTACTGGTAAGACTGAAACAACGTGTCTAAGGAACTACAATCCACTTACGGAGAATGATCTTGCTTTGCTTCAAGTTAATTCCTTACTCTCAATTTTCTTCCCAAATAGAGTCTTTCAGTGAAAAGCAGACTCCCGTAATATCCTGAACGTCTTCTTCGGTGTTATTTAACAGCAGGAACTGTGATTTTTAAACAATGGCTCTAGATTCA

**4. Primer designs for gene detection.**

| mRNA | Primer pairs |
| --- | --- |
| GAPDH (87bp)  (NM_001289726.1) | Forward: GGGTCCCAGCTTAGGTTCATC  Reverse: AATCCGTTCACACCGACCTT |
| TAPT1 (191bp)  (NM_173764.3)  RIC1 (130bp)  (NM_001081319.1)  FGF20 (199bp)  (NM_030610.2) | Forward: GCCAAGTATACAGAGCGAAGA  Reverse: CGGTCCCTTAAGCCATAGCAA  Forward: GGTACAGCCGACCTAGTGTG  Reverse: CCATTTGCTGTTGACACTGCT  Forward: CGCCGCATGTCTCTGGATAA  Reverse: CCATCTCAGTGTGGTGTGGT |
